# AQP3 as a potential biomarker for caries

**DOI:** 10.1186/s12903-026-08124-w

**Published:** 2026-03-25

**Authors:** Markus Baumann, Daria Pakosch-Nowak, Dominik Ziehe, Bjoern Koos, Andrea Witowski, Michael Adamzik, Jennifer Orlowski, Martin Kunkel, Katharina Rump

**Affiliations:** 1 Zahnarztpraxis Sprockhövel, Sprockhövel, Germany; 2 Department of Anesthesiology, Intensive Care Medicine and Pain Therapy, Ruhr University Bochum, Knappschaft Kliniken University Hospital Bochum, Bochum, Germany; 3 Department of Oral and Maxillofacial Surgery, Ruhr University Bochum, Knappschaft Kliniken University Hospital Bochum, Bochum, Germany

**Keywords:** AQP3, Saliva mRNA, Caries, Periodontitis, Biomarker, Risk factor

## Abstract

**Background and objectives:**

Dental caries and periodontitis are among the most common dental diseases worldwide and represent a major public health concern due to their high prevalence and age-dependent occurrence. Identifying molecular factors in saliva involved in their development may help improve prevention strategies. Aquaporins (AQPs), particularly aquaporin (AQP3)—a channel for water, glycerol and small molecules—have recently attracted scientific interest. This study aimed to investigate the association between salivary AQP3 expression and dental caries and periodontal parameters, evaluating its potential as a biomarker for oral health and disease.

**Materials and methods:**

The OKAPI study, a prospective observational study, enrolled 169 patients (middle Age: 45.54 ± 13.57; 37 % male) from a dental practice. Clinical parameters such as the periodontal screening index and Decayed, missing, filled teeth indices, along with demographic data, were recorded. Saliva samples were collected, RNA was isolated, and cDNA was synthesized. Quantitative RT-PCR was performed using primers specific for AQP3 and β-actin (ACTB). Results: Among the participants, 42% had severe caries and 12.4% had periodontitis. AQP3 mRNA level was significantly (p<0.05) decreased in patients with severe caries compared to unaffected individuals. An AQP3 level cut-off was calculated after receiver operating characteristic -analysis (area under the curve: 0.605) to distinguish between patients with and without severe caries. Subjects with low AQP3 levels had a higher caries prevalence. Logistic regression identified the AQP3 cut-off as a relevant, though age-dependent, risk factor.

**Conclusions:**

Reduced salivary AQP3 mRNA level was observed in individuals with severe caries and may indicate a potential association, suggesting that AQP3 could serve as a candidate biomarker for identifying individuals at increased risk.

## Introduction

Dental caries and periodontitis are among the most common dental diseases in Germany [1]. Their prevalence increases with age, making them a major public health concern. Globally, dental caries remains a widespread issue, with 2.5 billion people affected by untreated cavities in permanent teeth as of 2015 [2].

While current prevention strategies focus on diet and oral hygiene, they are not sufficient to fully prevent these diseases in certain high-risk populations [3, 4]. Further research is essential to identify molecular biological factors and biomarkers that could aid in developing more effective prevention approaches [5–8].

Saliva could depict an interesting medium for biomarker discovery, because of its easy, non-invasive accessibility and constant contact with teeth and periodontal tissues. Numerous studies have identified salivary biomarkers linked not only to oral but also to systemic diseases [9]. Our recent study confirmed the presence of aquaporin 3 (AQP3) mRNA in human saliva [10]. Aquaporin-3 (AQP3) is known to be expressed in epithelial cells of the oral mucosa, where it contributes to water and glycerol transport, barrier function, and inflammatory signaling. While the role of AQP3 has been more extensively studied in relation to periodontal disease and mucosal inflammation [11], its involvement in dental caries remains poorly understood.

Dental caries is primarily a demineralization disease driven by acid production from cariogenic bacteria in dental biofilms. However, epithelial integrity, mucosal immunity, and salivary composition also play important modulating roles in the caries process [12]. AQP3, being a key molecule in maintaining epithelial hydration and regulating responses to oxidative and inflammatory stress [13], could indirectly influence the caries process—for example, by affecting the local environment that supports or inhibits biofilm formation and microbial homeostasis.

AQP3 is a water channel protein mainly found in keratinocytes (cells of the skin and mucous membranes) [14, 15]. It facilitates not only water but also glycerol transport and has been implicated in inflammatory processes. Increased AQP3 expression has been observed in inflammatory gingival diseases such as periodontitis, where it may contribute to the regulation of the local immune response [14]. The precise role of AQP3 in caries remains speculative. It is plausible that AQP3 is linked to hyposalivation, which could serve as a potential mechanism for caries development, given that reduced salivary flow significantly hampers oral clearance [16]. In contrast to classical water-selective aquaporins, AQP3 belongs to the aquaglyceroporin family and additionally permits the transport of hydrogen peroxide (H₂O₂), thereby influencing intracellular redox signaling [17]. Importantly, AQP3 is also expressed in T cells, where AQP3-mediated H₂O₂ uptake regulates chemokine-dependent migration through activation of small Rho GTPases and actin cytoskeleton dynamics [18]. Given that progression of caries beyond dentin is associated with pulpal immune activation and T-cell infiltration, AQP3 is biologically positioned at the interface between epithelial barrier function, immune cell trafficking, and inflammatory signaling [19, 20]. Despite this mechanistic plausibility, the potential role of AQP3 in caries-associated immune responses has not been systematically investigated, representing a relevant knowledge gap.

Dental caries, on the other hand, is a disease caused by bacteria (mainly mutans streptococci) that produce acids from sugar, leading to demineralization of the tooth enamel. The main risk factors for caries are dental plaque, sugar intake, saliva flow, and the composition of the oral biofilm [21].

To date, there is limited evidence suggesting a direct association between Aquaporin-3 (AQP3) expression and the development of dental caries. AQP3 has primarily been studied in the context of inflammatory changes in the gingiva, such as periodontitis, rather than in relation to the demineralization processes characteristic of dental caries [14]. Although both caries and periodontitis are common oral diseases, they involve distinct pathophysiological mechanisms: caries is a demineralization-driven disease of the dental hard tissues, whereas periodontitis is an inflammatory disease affecting the supporting structures of the teeth.

While an indirect role of AQP3—for instance, via modulation of salivary flow or inflammatory pathways—cannot be ruled out, current research does not support a direct involvement of AQP3 in caries development. The present study therefore aims to explore a potential association between salivary AQP3 expression and caries as well as periodontal parameters, with the goal of evaluating its relevance as a possible biomarker in oral health and disease.

## Materials and methods

### Study design and cohort

The OKAPI study (German Clinical Trial Registry No. DRKS00032425, date of registration: 2023-08-16) prospectively enrolled patients meeting the inclusion criteria and was designed as an observational study. Approval for the study was obtained from the Ethics Committee of the medical faculty of the Ruhr University Bochum (23-7821-BR, approval date: 07.06.2023) and the Ethics Committee of the Westphalia-Lippe Medical Association (2023-416-b-S, approval date: 26.07.2023). The study protocol, site-specific informed consent forms, participant education and recruitment materials, and other required documents, along with any subsequent modifications, were reviewed and approved by these ethical review bodies. The study adhered to the revised Declaration of Helsinki, good clinical practice guidelines, and local regulatory requirements. Patients were recruited after providing written informed consent over a period of 17 months from 5th august 2023 to 31th december 2024 at a dental practice.

Inclusion and exclusion criteria:

Inclusion criteria were adult dental patients aged between 18 and 75 years (7.1% of the patients were 65 years or above). Exclusion criteria included genetically determined structural disorders of the dental hard tissue (e.g. amelogenesis imperfecta, dentinogenesis imperfecta, odontogenesis imperfecta), dementia and/or psychotic illness, lack of capacity to consent, and insufficient knowledge of the German language to understand the study’s scope and participation requirements.

Sample Size Calculation.

A total of 169 patients presenting for routine dental care were consecutively recruited for this study. Among these participants, 42% were classified as having severe caries, based on standardized clinical and radiographic criteria. Due to the lack of prior data on AQP3 expression in the context of dental caries, a formal sample size calculation for this study could not be performed a priori. The sample size was guided by our previous research in a comparable biomarker setting, where 87 septic patients were included to detect differences in AQP3 expression between survivors and non-survivors [22]. Given the exploratory nature of the present study, and to ensure sufficient power to detect meaningful differences in AQP3 expression between patients with severe caries and those with mild or no caries, we included a larger cohort of 169 participants. This approach provided adequately sized comparison groups for subsequent statistical analyses with a significance level of 0.05.

## Clinical Data

The primary outcomes of the study were to detect the prevalence of caries and periodontitis.

The prevalence of caries was detected by the Decayed, missing, filled teeth (DMFT) index.

When assessing the prevalence of caries, a patient is considered to have caries if at least one of the following criteria is met:

- Active dental caries on one or more teeth
- Missing tooth/teeth (extraction due to caries)
- Presence of one or more restored teeth by means of a filling or prosthetic restoration (e.g. dental crown)

In this study, we defined the severity of caries as follows:

- Very severe caries: 15 or more teeth meeting the above-mentioned criteria
- Severe caries: 10 to 14 teeth affected
- Medium caries: six to nine affected teeth
- Mild caries: five or fewer affected teeth

Periodontitis was defined as follows: To assess the gingiva s, the dentition is divided into six different sections (sextants [S1-6]) according to the Fédération Dentaire Internationale tooth numbering system:

- S1: Teeth 18-14
- S2: Teeth 13-23
- S3: Teeth 24-28
- S4: Teeth 38-34
- S5: Teeth 33-43
- S6: Teeth 44-48

Each section is examined with a periodontal probe, and the most severe code in the respective sextant determines the respective Periodontal Screening Index (PSI):

- Code 0: Probing depth < 3.5 mm, no bleeding on probing, no calculus, no protruding filling/crown margins
- Code 1: Probing depth < 3.5 mm, bleeding on probing, no calculus, no protruding filling/crown margins
- Code 2: Probing depth < 3.5 mm, calculus and/or protruding filling/crown margins
- Code 3: Probing depth 3.5-5.5 mm
- Code 4: Probing depth > 5.5 mm

The highest PSI index of all six sextants gives the overall result (0–4), which is interpreted as follows:

- Total code 0: Healthy
- Total code 1 or 2: Gingivitis
- Total code 3 or 4: Periodontitis

In addition to recording the caries and periodontitis status and collecting biosamples, clinical data and risk profiles were collected via a questionnaire. This were the secondary patient data. This survey included the following measurement variables:

- *Age*
- Gender (m/f/d)
- Pre-existing conditions: arterial hypertension, [non-]insulin-dependent diabetes mellitus
- Cardiovascular diseases (including CHD, post-myocardial infarction, PAD, etc.)
- Nicotine abuse (Current smokers smoked at least one cigarette per day at the time of examination; duration of smoking in years)Current or previous malignant tumor disease: localization, TNM stage, radio and/or chemotherapy
- Long-term medication (e.g. antiresorptive or immunosuppressive medication)

### Collection of samples

As part of the dental treatment in the dental practice, patients included in the study provided an oral mucosal swab and 2 ml saliva samples. The saliva samples were collected into Saliva RNA Collection and Preservation Devices (Norgen Biotek, BioCat, HD, Germany). These samples were then used to isolate RNA using the Total RNA Purification Kit (Norgen Biotek, BioCAT; HD, Germany). After isolation, the samples were stored at -80 °C for a maximum of a half year and were thawed on ice prior analysis. RNA quantification was performed using NanoDrop (Thermo Fisher, Ulm, Germany) device after storage.

### RNA quantification

The RNA was transcribed into cDNA and analyzed using qPCR for the analysis of candidate genes. For cDNA synthesis, 1 µg of RNA was used with the High-Capacity cDNA Reverse Transcription Kit (Thermo Fisher Scientific, Wilmington, USA). Specific primers (MWG eurofins, Ebersberg, Gerany) for AQP3 were used: AQP3_RT2_Se: 5′-GGAATAGTTTTTGGGCTGTA − 3′ and AQP3_RT2_As: 5′-GGCTGTGCCTATGAACTGGT − 3′. The qPCR reaction was carried out using GoTaq Mastermix (Promega, Walldorf, Germany). The levels were quantified using the ΔCt method, with β-Actin (ACTB) serving as the reference gene, as described previously [22, 23].

### Statistical Analysis

Patient characteristics are reported as percentages for categorical variables and as means with standard deviations (SD) or medians with interquartile ranges (25th and 75th percentiles), as appropriate. Categorical variables were compared using McNemar or Fisher’s exact tests. Continuous independent variables were compared using the Student’s t-test or the Mann–Whitney U test after testing for normal distribution with the Shapiro-Wilk Test. AQP3 mRNA level thresholds for discriminating patients with and without caries were determined via receiver operator characteristic (ROC) curve analysis. The Youden index was used to identify the point of best discrimination, which was then applied in a logistic regression analysis. Patients with missing data were excluded from analysis.

A binary logistic regression was performed to assess the relationship between age, the cut-off values, and the probability of caries. The logistic regression was conducted as follows: A logistic regression was performed using the data on age and disease status (yes/no) as well as whether the cut-off was exceeded or not. Then, the probability of being classified as diseased was calculated for different age values in both categories. Finally, the probability was plotted as a function of age, with separate curves for each category of the cut-off variable. The model was conducted without an interaction term to examine the independent effects of age and cut-off value on disease likelihood.

The p-values for age and cut-off value were calculated to determine the significance of their effects. A probability curve was generated for different age values, predicting the likelihood of disease for values above and below the cut-off. A line plot was created to visualize the relationship between age and disease probability, with a threshold line at 50% probability. The p-values for both age and cut-off value were reported in the plot title to indicate the significance of these variables.

A p-value of less than 5% was considered significant. Unless otherwise stated, data is always depicted as mean ± standard deviation (SD). All analyses were performed using SPSS (version 28, IBM, Chicago, IL, USA). GraphPad Prism 9 (GraphPad, San Diego, CA, USA) was used for graphical presentations.

## Results

In this study saliva samples from 169 patients were analyzed. The median age of the cohort was 46 years and 37.3% were male (Table 1). 42% of the patients had severe caries and 12.4% had periodontitis (Table 1).

**Table 1:** Baseline characteristics of the study cohort

Characteristics (*n* = 169)	Mean ± SD or N (%)	Minimum to maximum
Age	45.54 ± 13.57	18–74
Nicotine consume in years	4.3 ± 10.59	0–50
Gender male	63 (37.3%)	
Arterial hypertonia	25 (14.8%)	
Cardiac disease	8 (4.7%)	
Smoker	34 (20.1%)	
Diabetes	5 (3%)	
Cancer	3 (1.8%)	
- Bladder	- 1 (0.6%)	
- Mamma	- 2 (1.2%)	
Mild caries	38 (22.5%)	
Medium caries	60 (25.5%)	
Severe caries	71 (42.0%)	
Periodontitis	21 (12.4%)	

### AQP3 levels is decreased in patients with severe caries and periodontitis

**Fig. 1:**
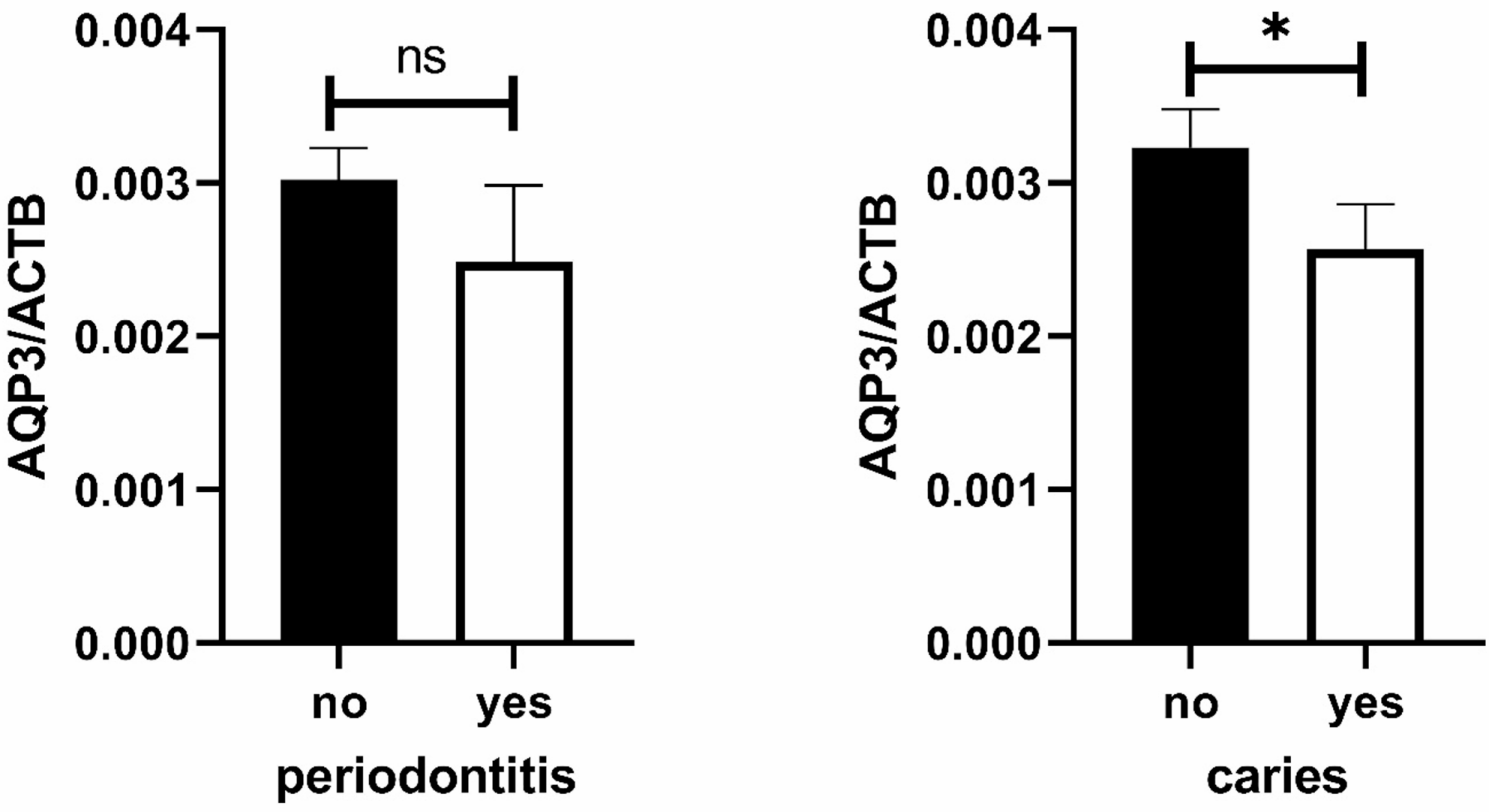
AQP3 mRNA levels in saliva samples of dental patients stratified by the occurrence of periodontitis (**a**, n= 169) or of severe caries (**b**, n=169) The quantity of AQP3 was normalized to the reference gene beta-actin (ACTB). ns = not significant; * p=0.0195

In the first step, AQP3 mRNA levels were measured in saliva samples. AQP3 mRNA level was found to be decreased in patients with severe caries compared to those with milder forms (*p* = 0.0195, Fig. 1b). Additionally, a tendency towards decreased AQP3 level was observed in patients with periodontitis compared to unaffected individuals (p = n.s., Fig. 1a).

### Determining of a cut off value for caries prediction

**Fig. 2:**
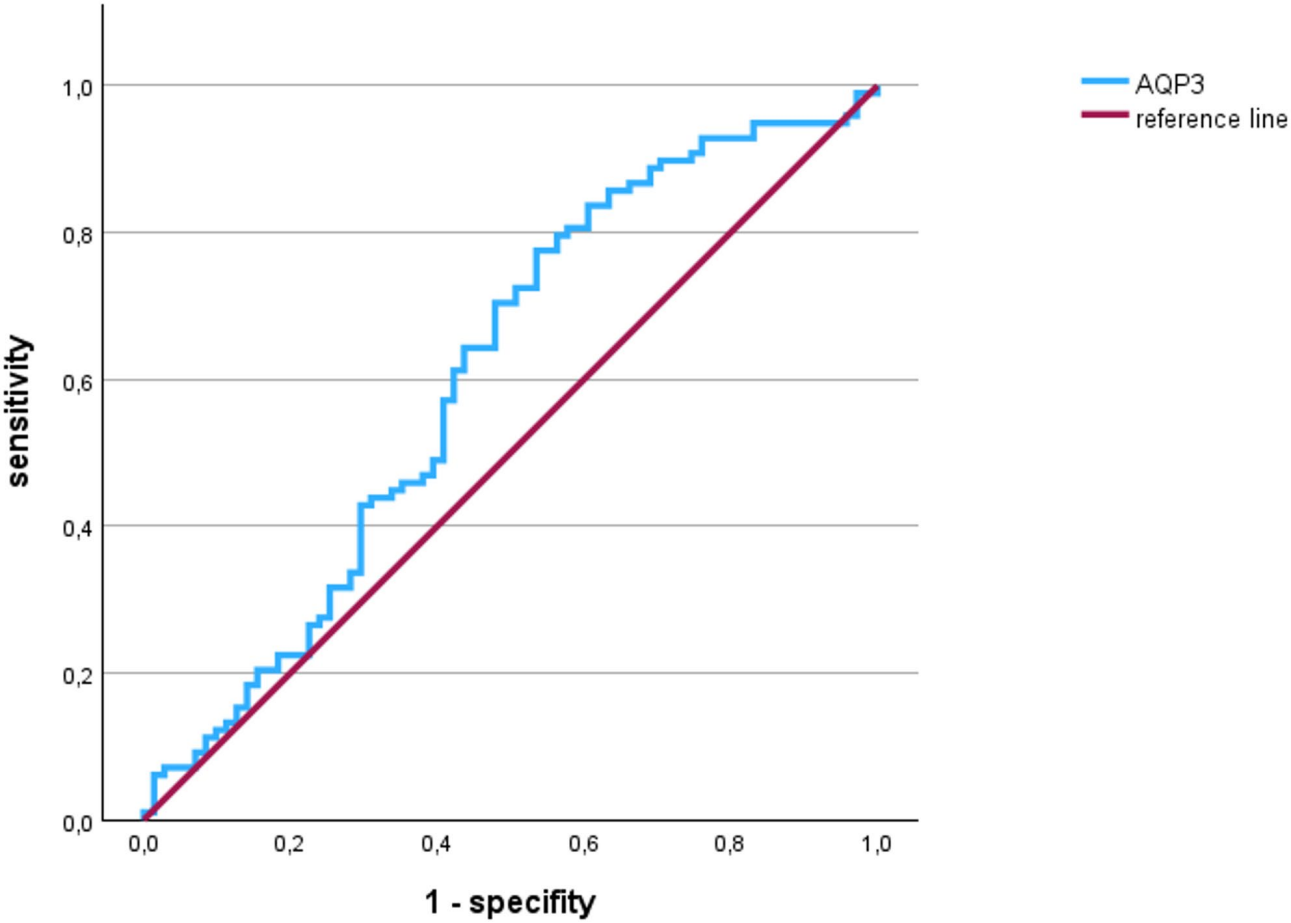
Receiver operating characteristic curve performed for AQP3 mRNA level and the occurrence of severe caries

ROC analysis was performed to determine the point of best discrimination between severe caries and non-severe caries, as well as between periodontitis and no periodontitis. The area under the curve (AUC) for caries prediction was 0.605 (95% CI: 0.516–0.694, *p* = 0.020, Fig. 2), and for periodontitis prediction, it was 0.431 (95% CI: 0.292–0.569, *p* = 0.326). Hence, no predictive value for periodontitis could be established. Using the Youden index, cut-off value was calculated, with a value of 0.001251 for caries.

Cross tables were used to detect differences in the patient groups with AQP3 levels below and above the cut-off for caries. The groups below and above the cut-off differed significantly only in the variable presence of severe caries (*p* = 0.002, Table 2).

**Table 2:** comparison in the baseline parameters between patients in group above and below cut-off using cross-tables. The groups were compared by Chi-square test

	Under cut-off (*n* = 56)	Above cut-off (*n* = 113)	*P*-value
Smoking (yes)	9 (16.1%)	25 (22.3%)	0.342
Arterial hypertonia (yes)	10 (17.9%)	15 (13.5%)	0.458
Cardiac disease	4 (7.1%)	4 (3.6%)	0.319
Diabetes	2 (3.6%)	2 (1.8%)	0.474
Cancer	1 (1.8%)	2 (1.8%)	1.000
Severe caries	33 (58.9%)	38 (33.6%)	0.002
Periodontitis	10 (17.9%)	11 (9.7%)	*0.132*
Age < 35	11 (19.6%)	32 (28.3%)	*0.149*
Age 35–50	16 (28.6%)	40 (35.4%)	
Age > 50	29 (51.8%)	41 (36.3%)	
Gender male	19 (33.9%)	44 (38.9%)	0.526

### Logistic regression analysis for the occurrence of caries

The binary regression analysis showed that the AQP3 cut-off value had the strongest effect on the development of caries with a hazard ratio of 2.794 (*p* = 0.012, Table 3), followed by age with a hazard ratio of 1.099 (*p* < 0.001).

**Table 3:** binary logistic regression model for the occurrence of caries

	*p*-value	Hazard ratio	95.0% CI	
			Lower	upper
AQP3 cut-off	0.012	2.794	1.257	6.210
Age	**< 0.001**	**1.099**	**1.058**	**1.141**
Gender male	0.169	0.587	0.274	1.254
Arterial hypertonia (yes)	*0.767*	0.857	0.309	2.379
Heart disease	0.097	0.192	0.027	1.344
Smoking (yes)	*0.730*	0.726	0.118	4.476
Smoking in years	0.905	1.004	0.937	1.077
Diabetes	1.000	0.000	0.000	0.000

**Fig. 3:**
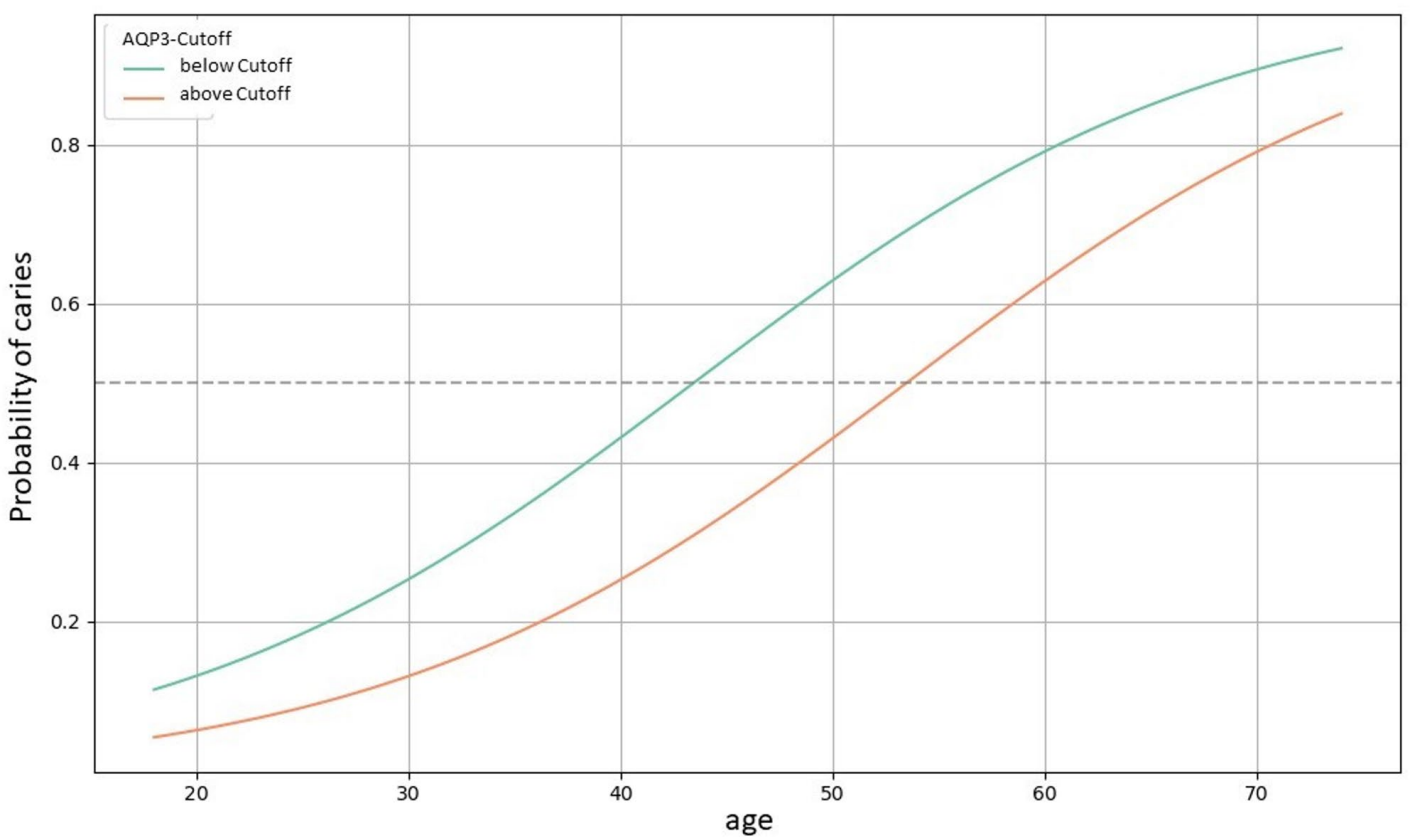
line graph for the probability of caries depending on age and stratified by the AQP3 cut-off value

Since the occurrence of caries appears to be age-dependent, a line graph was created to visualize the relationship between age and the probability of caries. The line graph shows how the probability of caries changes with age, separated by the categories of AQP3 cut-off values. For caries, falling below the cut-off is associated with a higher probability of developing caries (*p* = 0.0262, Fig. 3); however, age has the strongest effect (*p* < 0.0001, Fig. 3). We wondered whether AQP3 level is correlated to age and in the overall cohort of dental patients, we observe a negative correlation with age (*r* = − 0.176, *p* = 0.022). However, when splitting the cohort into patients without caries (*r* = − 0.08, *p* = 0.415) and with caries (*r* = − 0.16, *p* = 0.181), this correlation is no longer evident.

## Discussion

Our study demonstrated a reduced level of Aquaporin-3 (AQP3) in the saliva of patients with caries and periodontitis. Furthermore, diminished levels of AQP3 appear to be a risk factor for the development of caries, with age also playing a significant role. To the best of our knowledge, this is the inaugural study to explore the involvement of AQP3 in caries.

Our study is the first to demonstrate an association between salivary AQP3 mRNA expression and chronic periodontitis, highlighting its potential as a non-invasive biomarker reflecting epithelial and immune alterations in the oral inflammatory microenvironment.

Existing evidence on acquired risk factors for caries, predominantly derived from pediatric studies, underscores hyposalivation, smoking, and certain medical conditions as contributing factors. Contrary to these findings, our study did not identify smoking as a pertinent risk factor for caries development. Hyposalivation, which can result from medications, radiation therapy, or Sjögren’s syndrome, along with maternal smoking and poorly controlled type 1 diabetes, has been associated with an elevated risk of caries, although the evidence supporting these associations is generally weak [24].

In our study, we observed that decreased AQP3 mRNA levels in saliva was significantly associated with higher caries experience. This novel finding suggests a potential protective role of AQP3 in maintaining oral health. One possible explanation is that reduced AQP3 levels may impair epithelial barrier function or alter local immune responses, thereby facilitating the proliferation of cariogenic bacteria and acid-induced demineralization.

To our knowledge, this is the first study to demonstrate an inverse association between AQP3 levels and caries severity, highlighting the need for further research into the molecular mechanisms linking epithelial water channels to caries pathogenesis. Future studies should explore whether AQP3 could serve not only as a biomarker but also as a potential therapeutic target in caries prevention. Our findings suggest that salivary AQP3 has potential as a biomarker for dental caries, although its predictive performance is moderate. The area under the curve (AUC) for AQP3 in discriminating individuals with severe caries from those with mild or no caries was 0.605, indicating only modest discriminatory ability. However, the hazard ratio (HR) derived from binary logistic regression was 2.79, demonstrating that higher AQP3 expression is associated with a substantially increased likelihood of severe caries. This effect size is comparable to those reported for other salivary biomarkers in previous studies, which range from OR = 2.1–3.2 for proteins such as statherin or HNP1–3, and AUC values up to 0.926 for composite biomarker [25]. While AQP3 alone does not reach the high predictive accuracy of some of these markers, its integration into multi-marker panels could improve predictive performance and provide mechanistic insights into the interplay between epithelial function, immune cell trafficking, and caries development. Therefore, AQP3 represents a promising candidate for further investigation as part of a broader salivary biomarker strategy.

In addition, given that AQP3 is highly expressed in immune cells, other immunological mechanisms may also play a role. To our knowledge, only two other studies have investigated the potential role of AQP3 in oral diseases. Interestingly, while previous studies have reported increased salivary AQP3 in conditions such as xerostomia, we observed reduced AQP3 expression in patients with periodontitis [26]. Patients with periodontal disease exhibited a high prevalence of subjective xerostomia despite normal unstimulated salivary flow rates, and salivary AQP3 protein concentration was higher in those with xerostomia, suggesting its potential as a biomarker [26]. Here, the aforementioned results (lower AQP3 level in periodontitis) would also contradict the literature (AQP3 increase in inflammation and xerostomia). These contradictions cannot be fully clarified with the present study. Nevertheless, this discrepancy likely reflects the different pathophysiological contexts: in xerostomia, elevated AQP3 may represent a compensatory response of the salivary glands to maintain water and glycerol transport despite reduced salivary flow. In contrast, chronic inflammation in periodontitis can disrupt epithelial integrity and downregulate transport proteins, including AQP3, potentially impairing local fluid homeostasis and contributing to tissue inflammation. These findings suggest that AQP3 expression is dynamically regulated in response to oral stress, and that reduced AQP3 may serve as a marker of epithelial and immunological dysfunction in periodontitis, rather than being a direct cause of disease [14].

However, AQP3 seems to be expressed in salivary glands, but there is not much known about the expression and function of AQP3 in them [27]. AQP3 was detected at the apicolateral membranes of both mucous and serous acini throughout the stages of human salivary gland morphogenesis. This suggests that AQP3 plays a significant role in the maturation of human salivary glands, with distinct expression patterns indicating its involvement in glandular development [28]. Additionally, AQP3 similar to AQP5 could potentially influence fluoride levels in saliva, thereby affecting remineralization processes. Such a regulatory mechanism would still need to be demonstrated. As currently it was observed that AQP5 expression decreases with increased fluoride availability, but it was not shown that, conversely, AQP5 expression regulates fluoride availability [29]. One other possibility is the ability of AQP3 to transport glycerol [30]. Thus, it could regulate glycerol levels in saliva, which could influence the metabolic activity of cariogenic bacteria such as Streptococcus mutans [31]. Glycerol is a substrate that can be metabolized by these bacteria and contributes to the production of acids that demineralize tooth enamel [32].

In addition, the role of AQP3 in immune regulation may be critical for defense against caries-associated pathogens [13]. AQP3 is primarily found in T-lymphocytes [17]. T-Lymphocyte deficiency, particularly of T-cytotoxic lymphocytes/suppressors, plays a pathogenetic role in the development and progression of dental caries in young people who have suffered from coronavirus disease [33]. The prevalence of dental caries was high in HIV-infected children on antiretroviral therapy, particularly in those with advanced CD4 count stages, with a decrease in absolute lymphocyte count [34]. Children born to HIV-infected mothers are at a higher risk of dental disease, with perinatal HIV infection significantly altering the oral microbiota, particularly in those with low CD4 levels, which seems to be contrary to the above mentioned study. While HIV infection has a lasting impact on the oral microbiome, the effect of perinatal exposure without infection (HIV-exposed-but-uninfected children) appears to be transient [35]. The data of the role of T-cells in caries development seem to be limited. As an example natural, interleukin-17 (IL-17)-producing gamma-delta T cells (γδT, Th17) are abundant innate immune cells that can act as effectors without explicitly inducing an immune response. Th17 cells contribute to inflammatory diseases, including periodontitis, by promoting gingival inflammation and bone destruction, but there is currently no evidence for a role of IL-17 in caries [19].

Thus, by modulating inflammatory responses in the oral cavity, AQP3 may influence the balance between protective and pathogenic factors in dental biofilms [32, 36]. Understanding these mechanisms may open new avenues for preventive and therapeutic strategies targeting AQP3 in saliva.

A limitation of the present study is that it does not experimentally demonstrate how reduced AQP3 expression directly contributes to caries development. While we observed an association between higher salivary AQP3 levels and increased risk of severe caries, the underlying mechanistic link remains unclear. It is possible that AQP3 influences caries susceptibility indirectly, for example by modulating salivary composition, epithelial barrier function, or immune cell trafficking, rather than directly affecting demineralization processes. Future studies employing functional assays, such as in vitro models of AQP3-mediated water and glycerol transport or manipulation of AQP3 expression in salivary epithelial cells, are needed to clarify its causal role and to determine whether AQP3 could be a target for preventive or therapeutic interventions.

Limitations of or study should be mentioned. First, potential confounders such as salivary flow rate, diet, and smoking were not controlled for, which may have influenced salivary AQP3 expression and caries outcomes. Second, the study focused solely on qPCR analysis of AQP3 mRNA in saliva; no protein-level assessments or functional assays were performed. Third, the study does not clearly describe participant diversity (e.g., age groups, health status, oral hygiene habits), which limits the generalizability of our findings to broader populations. In further analyses it would be helpful to use immunohistochemistry or Western Blot to detect AQP3 protein expression in salivary glands and saliva samples. In addition functional assays to test water and glycerol permeability of AQP3 in vitro could be of interest [37]. Future studies could also focus on developing specific AQP3 enhancers or inhibitors to evaluate their effects on salivary composition and function [38, 39]. Further studies are needed to clarify the exact function and therapeutic potential of AQP3 in saliva.

## Conclusions

In conclusion, this study found that low AQP3 level in saliva is associated with a higher prevalence of caries and periodontitis. This finding suggests that AQP3 may represent an intriguing biomarker or target for oral health. Further research is warranted to explore the potential of AQP3 in diagnostic and therapeutic applications, which could significantly enhance the management and prevention of these prevalent oral diseases.

## Data Availability

The datasets used and/or analysed during the current study are available from the corresponding author on reasonable request.

## Abbreviations

AQP: Aquaporin
SD: Standard deviation
RNA: Ribonucleic acid
DNA: Deoxyribonucleic acid
PSI: Periodontal Screening Index
